# Design of a multicentre randomized controlled trial to evaluate the effectiveness of a tailored clinical support intervention to enhance return to work for gastrointestinal cancer patients

**DOI:** 10.1186/s12885-016-2334-x

**Published:** 2016-05-10

**Authors:** AnneClaire G.N.M. Zaman, Kristien M.A.J. Tytgat, Jean H.G. Klinkenbijl, Monique H.W. Frings-Dresen, Angela G.E.M. de Boer

**Affiliations:** Coronel Institute of Occupational Health, Academic Medical Center, University of Amsterdam, Amsterdam, The Netherlands; Gastrointestinal Oncological Center Amsterdam, Academic Medical Center, University of Amsterdam, Amsterdam, The Netherlands; Department of Surgery, Gelre Hospital, Apeldoorn, The Netherlands; Faculty of Medicine, University of Amsterdam, Amsterdam, The Netherlands

**Keywords:** Return to work, Employment, Sick leave, Psycho-oncological care, Oncological occupational physician, Gastrointestinal cancer

## Abstract

**Background:**

Gastrointestinal (GI) cancer is frequently diagnosed in people of working age, and many GI cancer patients experience work-related problems. Although these patients often experience difficulties returning to work, supportive work-related interventions are lacking. We have therefore developed a tailored work-related support intervention for GI cancer patients, and we aim to evaluate its cost-effectiveness compared with the usual care provided. If this intervention proves effective, it can be implemented in practice to support GI cancer patients after diagnosis and to help them return to work.

**Methods/Design:**

We designed a multicentre randomized controlled trial with a follow-up of twelve months. The study population (*N =* 310) will include individuals aged 18–63 years diagnosed with a primary GI cancer and employed at the time of diagnosis. The participants will be randomized to the intervention or to usual care. ‘Usual care’ is defined as psychosocial care in which work-related issues are not discussed. The intervention group will receive tailored work-related support consisting of three face-to-face meetings of approximately 30 min each. Based on the severity of their work-related problems, the intervention group will be divided into groups receiving three types of support (A, B or C). A different supportive healthcare professional will be available for each group: an oncological nurse (A), an oncological occupational physician (B) and a multidisciplinary team (C) that includes an oncological nurse, oncological occupational physician and treating oncologist/physician. The primary outcome measure is return to work (RTW), defined as the time to a partial or full RTW. The secondary outcomes are work ability, work limitations, quality of life, and direct and indirect costs.

**Discussion:**

The hypothesis is that tailored work-related support for GI cancer patients is more effective than usual care in terms of the RTW. The intervention is innovative in that it combines oncological and occupational care in a clinical setting, early in the cancer treatment process.

**Trial registration:**

METC protocol number NL51444.018.14/Netherlands Trial Register number NTR5022. Registered 6 March 2015.

## Background

Patients with gastrointestinal (GI) cancer experience work-related problems at the time of diagnosis [1]. In general, cancer survival rates have been improving in recent years due to early detection via screening programmes and continuous improvements in treatment [2, 3]. The burden of the disease itself and the treatment not only negatively affects quality of life [4–6] in all its aspects, but also work participation [7], with the additional financial consequences this brings [8, 9]. Work participation is becoming increasingly important in today’s ageing society, meaning that the incidence of cancer in patients of working age is also increasing. Furthermore, the raising of the retirement age is expected to bring an additional increase in the number of cancer survivors in the working population.

It is important that cancer patients are able to return to work (RTW), because having a fulfilling work life is associated with a higher quality of life [10, 11] and provides much-needed income [12]. Most cancer survivors want to resume work after treatment, but regrettably, not all survivors are able to do so, because they experience various difficulties and treatment-related factors [1, 13, 14], or even become unemployed [15]. In addition to diagnosis and treatment, patients have to deal with physical, emotional and social problems such as fatigue, pain, cognitive deficits, anxiety and depression. These can impair social functioning and affect the patients’ ability to RTW [15, 16].

Relatively few studies have assessed the employment status of patients with GI cancer [17, 18] compared to well-studied diagnoses such as breast cancer [19, 20]. Besides work-related problems due to cancer, GI cancer patients can be confronted with specific GI cancer-related problems, such as eating, defecation or stoma problems, which can interfere with their work and for which they need support. Work-related problems are experienced both at the time of diagnosis and during treatment, but no work-related support (in the form of interventions) is provided nowadays in curative settings. Earlier research has shown that patients appreciate receiving information on their RTW in the early stages of cancer treatment [1, 21, 22]. It is therefore essential to have an in-hospital-based care programme to support the RTW process. Moreover, work-related problems can vary in severity, meaning that any such intervention should provide tailored support.

We have developed a tailored work-related support intervention for GI cancer patients. We aim to evaluate the cost-effectiveness of the tailored work-related support intervention as compared with usual care. If this intervention proves effective, it can be implemented in practice to support GI cancer patients, starting at diagnosis, during treatment and in the period after diagnosis and treatment to help them RTW.

### Objective

The objective of this paper is to present the design of a study that aims to evaluate the tailored work-related support intervention in terms of cost-effectiveness.

## Methods/Design

**Fig. 1:**
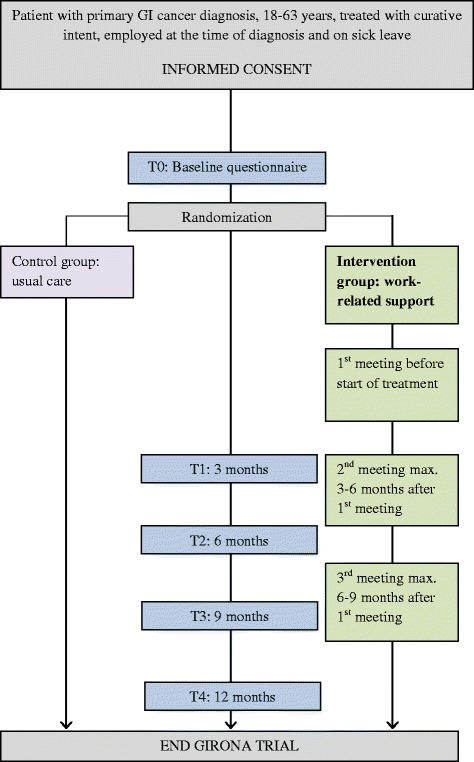
Flow chart of RCT, design of the GIRONA study

This study will take the form of a two-armed non-blinded multicentre randomized controlled trial (RCT), with a follow-up of twelve months (see Fig. 1). We will compare the tailored work-related support intervention with the control group receiving the usual care, which takes the form of standard psycho-oncological care. Data will be gathered using questionnaires at baseline, three, six, nine and twelve months. The CONSORT statement was used to report the design of this study [23].

The Medical Ethical Committee of the Academic Medical Center Amsterdam, the Netherlands, approved the study. The local medical ethics committees of the participation hospitals gave positive advice on the local feasibility of the study. The participating hospitals are Albert Schweitzer hospital Dordrecht, Amphia hospital Breda, Gelre hospitals location Apeldoorn and Zuthpen, Martini hospital Groningen, the University Medical Center Groningen, Meander Medical Center Amersfoort, OLVG hospital Amsterdam and the Medical Center Alkmaar.

METC protocol number NL51444.018.14

Trial registration number NTR5022 registered on 6 March 2015.

### Study population

The study population (*N =* 310) will include individuals aged between 18 and 63 years, with a primary diagnosis of GI cancer (oesophagus, stomach, liver, pancreas, biliary, small intestine, colon or rectum cancer), treated with curative intent (no study restrictions for type of treatment), employed at the time of diagnosis (including self-employment) and being on sick leave as a result of work-related problems due to the cancer diagnosis. The exclusion criteria are: 1) patients who are unable to speak, read or write Dutch sufficiently; 2) patients who have a severe mental disorder or other severe co-morbidity; and 3) patients who are receiving primary treatment at another hospital.

### Recruitment of participants

Eligible patients will be recruited between May 2015 and July 2016. GI cancer patients will be asked to participate at the hospital where they are receiving their treatment. The oncologist or oncological nurse will check each patient’s eligibility by assessing the inclusion and exclusion criteria. During their first visit to the hospital where they are being treated, patients will be informed about the study by their oncologist or oncological nurse. The oncologist or oncological nurse will then provide the patient with a brief explanation of the study and the patient will be asked whether the researcher may contact them by telephone. If the patient agrees, he or she will sign a specific informed consent form for telephone contact. The patient will be given a folder with an information leaflet, contact information and the informed consent form. If the patient immediately decides not to participate, the oncologist or oncological nurse asks the patient whether he/she wants to provide a reason for not participating, but the patient is not obliged to do so. If the patient has given their informed consent to be contacted by telephone, the researcher will phone the patient within one week. A meeting (in person or by telephone) will be scheduled at in which outstanding questions relating to the study can be answered, and at which the informed consent form will be signed in the presence of the researcher before the patient can participate in the study (which is known as GIRONA: GastroIntestinal cancer patients Receiving Occupational support Near and After diagnosis). The patient subsequently receives the baseline questionnaire, either on paper or digitally (depending on their preference). If they do not return the baseline questionnaire, the researcher will contact them by telephone after one week.

Participants can leave the study at any time, for any reason, without any consequences for their normal cancer care. They will be asked about their reason for withdrawal, but they will not be obliged to answer this question.

### Randomization

When the researcher has received the baseline questionnaire, the patient will be randomized to 1) the intervention group that will receive tailored work-related support, or 2) the control group that will receive care as usual. A computerized randomization programme ALEA [24] will be used to do this. As patients differ between the participating hospitals in terms of diagnosis, demographic factors and age, and because these factors are important prognostic factors for RTW [25], randomization will be stratified for gender, age (age groups: 18–54 and 55–63 years) and hospital, so as to prevent bias due to unequal randomization. Minimization will be applied to equalize group sizes. The patients, healthcare professionals and researchers will not be blinded for the group assignment.

### The intervention: tailored work-related support

The intervention consists of three individual meetings between a patient and a healthcare professional within the clinical setting. As work-related problems differ in severity, the intervention will be divided into three types of support, namely: support A, support B and support C.

In an earlier study (published separately) with an expert panel consisting of physicians, oncological nurses, (oncological) occupational physicians, social workers and patients, a decision diagram was drawn up that, based on a list of factors from the literature, refers the patient to one of the three types of support (A, B or C) for the tailored work-related support intervention (see Table 1). These factors have been added to the patients’ baseline questionnaire (T0). Based on a participant’s answers to this baseline questionnaire and the decision diagram, the researcher will refer the participant to the tailored types of support A, B or C.

**Table 1:** Overview of tailored support (A, B and C), depending on type of work-related problems experienced

Healthcare professional	A (oncological nurse)	B (occupational physician)	C (multidisciplinary team)
Factors (differ according to type of support)	• Fatigue • Pain • Treatment • Uncertainty about the future • Lack of support from family and friends	• Lack of support in work environment • Neuropsychological symptoms • Occurrence of side effects that prevent patient from doing current work *Combination of the following factors:* Number of working hours—treatment—stage of cancer and fatigue	*Combination of the following factors:* Medical complications—fatigue—stage of cancer—type of cancer—perception of change regarding work—dietary problems
First meeting Points for discussion include:	• Discuss restrictions on work due to cancer • Inform patient about the importance of work • Feasibility of continuing to work during treatment • Patient’s point of view on work • Plan to RTW or sustain work	• Discuss restrictions on work due to cancer • Inform patient about the importance of work • Feasibility of continuing to work during treatment • Patient’s point of view on work • Plan to RTW or sustain work • Advice on workload/planning working hours/tasks, etc.	• Preliminary interview, occupational physician and patient • Multidisciplinary team consultation • Occupational physician provides feedback to patient • Discuss restrictions on work due to cancer • Inform patient about the importance of work • Feasibility of continuing to work during treatment • Patient’s point of view on work • Plan to RTW or sustain work • Advice on workload/planning working hours/tasks, etc.
Second meeting Points for discussion include:	• Discuss contact with work environment • Already resumed work or still working • Barriers to RTW • Discuss key decision-points within the process of reporting sick leave (legal obligations) • Evaluate aims first meeting	• Discuss contact with work environment • Already resumed work or still working • Barriers to RTW • Discuss key decision-points within the process of reporting sick leave (legal obligations) • Evaluate aims first meeting	• Discuss contact with work environment • Already resumed work or still working • Barriers to RTW • Discuss key decision-points within the process of reporting sick leave (legal obligations) • Evaluate aims first meeting
Third meeting (if indicated)	Repeat matters discussed in second meeting • Discuss contact with work environment • Already resumed work or still working • Barriers to RTW • Discuss key decision-points within the process of reporting sick leave (legal obligations) • Evaluate aims first and second meeting	Repeat matters discussed in second meeting • Discuss contact with work environment • Already resumed work or still working • Barriers to RTW • Discuss key decision-points within the process of reporting sick leave (legal obligations) • Evaluate aims first and second meeting	Repeat matters discussed in second meeting • Discuss contact with work environment • Already resumed work or still working • Barriers to RTW • Discuss key decision-points within the process of reporting sick leave (legal obligations) • Evaluate aims first and second meeting

The three support types also differ in terms of the healthcare professional providing the supportive care. The intervention will be implemented by an oncological nurse in support type A, by an oncological occupational physician (specialized in oncological patients’ RTW) in support type B, and by a multidisciplinary team (including, at a minimum, an oncological nurse, the treating physician and the oncological occupational physician) in support type C. The main differences in the three types of support within the intervention are thus the factors for which a patient is referred to support type A, B or C, and the healthcare professionals who provide the support (Table 1).

Before the start of the study, the oncological nurses who will give the work-related support in the intervention participate in a training session lasting 2.5 h. This training session will consist of an informative part on the GIRONA study, the intervention and the importance of work for patients, and training on the rights and obligations of employees on sick leave in the Dutch social security system. Furthermore, work-related problems due to cancer and cancer treatment will be discussed, such as the feasibility of continuing to work during treatment, the factors that have an impact on the RTW, and the extent to which patients are open about their cancer with colleagues and employers. In addition, the checklist forms, which serve as a guideline for conversation, are discussed and practised with reference to a case.

The training session is given by the researcher [AZ], who has clinical experience as a nurse and conducts research on cancer and work. The training session includes elements that were used in previous research, in which a work-directed intervention was developed consisting of four meetings with a nurse and a meeting with the occupational physician and supervisor to make a RTW plan [26]. The oncological occupational physicians are specialized in supporting patients with cancer who encounter work-related problems. They followed an accredited training course on ‘basic training for oncological occupational physicians’, given by the Netherlands School of Public and Occupational Health (NSPOH) in Amsterdam, the Netherlands.

The aim of the *first meeting* is to inform patients about the importance of work during and after treatment, to identify any work-related problems, and to make a plan for the RTW (Table 1). The meeting will be scheduled before the start of the treatment and will take the form of an individual face-to-face meeting lasting approximately 30 min.

The *second meeting* aims to inform and evaluate the goals of the first meeting. This meeting will be scheduled after the first meeting, in consultation with the patient and the supporting healthcare professional (depending on the diagnosis/treatment and preferences of the patient). It will take place no more than three to six months after the first meeting. It will take the form of an individual face-to-face meeting and last about 30 min (Table 1). At the end of the meeting, the healthcare professional will evaluate whether the work-related support being provided is sufficient (on the basis of the decision diagram and the patient’s answers). If the support proves to be insufficient, the type of support will be adjusted.

The *third meeting* will consider the same issues as the second meeting: evaluating the goals of the first and second meetings. This meeting will also be scheduled at the request of the patient and/or the supporting healthcare professional at the second meeting, depending on diagnosis/treatment and the preferences of the patient, and in a maximum of six to nine months after treatment (Table 1).

In the support C-type intervention (multidisciplinary meeting), the oncological occupational physician will first hold a face-to-face meeting with the patient before the multidisciplinary meeting. The team will subsequently confer to discuss the outcomes identified by the oncological occupational physician with respect to RTW possibilities and restrictions. The oncological occupational physician will lead the multidisciplinary team consultation and is designated to provide the patient with feedback in a further face-to-face or telephone meeting.

### Care as usual

Care as usual in the clinical setting involves standard psychosocial care provided by the oncological nurse during regular appointments. In most cases, this means that the patient’s perceptions after treatment and possible treatment-related problems or complications are discussed. However, RTW, work-related problems or treatment-induced work problems are not routinely discussed in this form of psychosocial care.

### Sample size

The calculation of the sample size is based on two earlier studies, because no recent data are available on the RTW of GI cancer patients from clinical patient cohorts. Data from 2005 on 220 GI cancer patients, provided by a large occupational health service in the Netherlands, show that two years after diagnosis, 63 % of patients had returned to work full-time [27]. Based on these data, it is hypothesized that the percentage of 63 % RTW in GI cancer patients is the maximum result of ‘care as usual’. In a study by Nieuwenhuijsen [28], an intervention group of 35 radiotherapy patients received a supportive work-related intervention in a hospital. RTW was measured between the end of curative treatment and the first day of (partial) work resumption. This was found to be 89 % after twelve months. The median time between enrolment and the end of treatment was 42 days. Earlier studies at the AMC show that the percentage of patients that RTW in the first 42 days is around 8 % [29, 30]. The RTW for the intervention group was therefore set at 89 %–8 % = 81 %.

The sample size is calculated using the Power and Sample Size Calculation program nQuery Advisor 7.0. A power of 80 % and a p value of <0.05 indicated that we should include a total of 216 patients, with a follow-up of twelve months, to indicate a difference of 81 % (intervention) versus 63 % RTW (care as usual). Allowing for a 20 % loss to follow-up and a 10 % one-year mortality rate, 309 patients should be included.

### Outcome measures and prognostic factors

Data on the primary and secondary outcomes, as well as prognostic factors such socio-demographic factors (i.e. age, gender, level of education), disease-related factors and work-related factors, will be gathered using five questionnaires: baseline (T0) and three (T1), six (T2), nine (T3) and twelve (T4) months after baseline. It will take approximately 30 min to complete a questionnaire. The primary outcome measure is RTW, defined as the time to a partial or full RTW, and measured as the number of calendar days between the first day of sick leave and the first day at work. The patient must have returned to work (part-time or full-time) for at least four successive weeks (independent of the number of contract hours). The secondary outcome parameters are work ability, work limitations, quality of life, RTW (yes/no) and direct/indirect costs.

#### Effect evaluation

The effectiveness of the study will be determined on the basis of the primary outcome RTW and the secondary outcomes work ability, work limitations and quality of life, assessed at a long-term follow-up at twelve months. RTW will be based on the patients’ self-reporting in the questionnaires (T0-T4).

Work ability will be assessed with reference to the first three questions of the Work Ability Index (WAI) [31], using a ten-point scale. These questions concern an evaluation of a patient’s current work ability compared to their lifetime best ability, and their current physical and mental work ability with respect to their job demands. Higher scores indicate a higher level of work ability (0 means not currently able to work at all, and 10 means work ability at its best). The index has been assessed as having good levels of reliability and validity [32].

Work limitations will be measured with the Work Limitation Questionnaire (WLQ) [33], using a five-point scale. This questionnaire evaluates work disability and productivity (loss) in people with health problems. It consists of four sub-scales with 25 items: work scheduling, physical demands, mental/social demands and output demands. The scale ranges from 0 to 100, with higher scores indicating more work limitations over the previous two weeks (0 means never limited and 100 means limited all of the time). The English version of the WLQ has been shown to be valid and reliable when used for cancer survivors [34]. The WLQ questionnaire has been translated into Dutch and is valid and reproducible at group level among cancer survivors [35].

Quality of life will be measured with two questionnaires; 1) the SF12 [36, 37], to compare the study participants with the normal population, and 2) the EORTC QLQ-C30 [38], to compare the quality of life of the study participants diagnosed with GI cancer with that of patients with other cancer diagnoses. These questionnaires provide insight in the participants’ feelings about their health and their ability to carry out their usual activities.

The SF12 consists of twelve items, providing scales for physical and mental health. Questions refer to the patient’s functional status, including their physical and social functioning and physical and emotional constraints and their general view of their own health. The physical and mental health scores are calculated using the scores of twelve questions. The questionnaire has a range from 0 to 100 (0 means the lowest level of health measured by the scales, and 100 indicates the highest level of health).

The EORTC QLQ-C30 contains 30 items, including five functional scales, three symptom scales, a global health status/Quality of Life scale and six single items. Scores range from 0 to 100. A higher score represents a higher (better) level of functioning or a higher (worse) level of symptoms.

#### Process evaluation

The process of the intervention will be evaluated with the following parameters: number of consultations within the intervention, number of consultations with other work-related support interventions, and participant satisfaction, measured with the self-reporting questionnaires. Participants may use any co-interventions that they wish to use; there will be no restrictions. In order to evaluate this, participants in both the intervention and the control groups will be asked in the self-reporting questionnaires about the type and number of co-interventions they received.

The content of the consultation, the number of referrals to the other types of support within the intervention, the evaluation (using the decision diagram) of whether the work-related support is sufficient and the health professionals’ general perceptions of and satisfaction with the intervention are measured using the protocol forms, which are completed during the meeting with the patient.

#### Economic evaluation

A cost-effectiveness analysis (CEA) will be performed, taking a societal perspective. The economic evaluation will cover work-related costs to society and the cost of healthcare for the patients themselves and for their employers. The following costs (payment discipline for intervention meetings/nurse training/days of patient’s hospital admission/patients’ income and work adjustments) will be measured for the intervention group as well as for the control group.

Indirect costs, including absenteeism (determined as the total number of days of sick leave from the first day of sick leave at baseline until follow-up), lost earnings (determined as the difference in income between baseline and follow-up) and work productivity (measured using the Work Limitation Questionnaire), will be taken into account and obtained with the patients’ questionnaires (T0-T4). Direct costs such as the duration of the intervention (in minutes) for each patient by the healthcare professional will be presented. The healthcare professionals will record the duration of each meeting.

#### Prognostic factors

The prognostic factors include age, gender, marital status (married/single/cohabiting/widowed/divorced), education (seven categories), diagnosis (primary GI cancer diagnosis: oesophagus, stomach, liver, pancreas, biliary, small intestine, colon or rectum), treatment type (surgery, radiotherapy, chemotherapy, hormonal therapy, other) and treatment duration assessed with the self-reporting questionnaires. Fatigue is measured using the Multidimensional Fatigue Inventory questionnaire (MFI) [39], with 20 items divided into five sub-scales: general fatigue, physical fatigue, reduced activity, reduced motivation and mental fatigue. The sub-scale scores range from 4 to 20; a higher (implies 20) score indicates greater fatigue. Depression is measured using the scale developed by the Centre for Epidemiologic Studies for Depression (CES-D) [40], scored with 20 items. The scores range from 0 to 60; a higher (implies 60) score indicates greater depressive symptoms weighted for the past week. Cognitive functioning is measured with the Cognitive Symptom Checklist-Work, Dutch Version (CSC-W DV), containing 19 items, with the sub-scales for working memory and executive function. Scores range from 0 to 100, with higher scores indicating more cognitive symptoms (i.e., more limitations) in the work context. This questionnaire has been translated and a customized version of the Cognitive Symptom Checklist-Work (CSC-W) [41] validation article will be published.

#### Descriptive factors

The descriptive factors include breadwinner status (yes/no), job position (permanent contract/temporary contract/temporary worker/self-employed/other), employment status (employed/unemployed/self-employed/pension), years in current position, and company size (large 100 ≥ employees/medium-sized 10–100 employees/small 1–10 employees, or not applicable; self-employed), which will be assessed with the self-reporting questionnaires. The stage of cancer will be assessed from the patient’s medical records. Furthermore, the Perception and Judgement of Work (VBBA) [42] questionnaire will be used, with the sub-scale ‘physical workload’ containing seven items. The scores range from 0 to 21; a higher score indicates a higher physical workload. The importance of work is measured with a Visual Analogue Scale (VAS-scale), with a line running from 0 (not important) to 10 (most important).

### Statistical analysis

All analyses will be conducted in accordance with the intention-to-treat (ITT) principle. After randomization, participants will be labelled with a research code consisting of a unique number. This unique number will be used for the data analysis, to guarantee blind analysis of the data by the researcher. Baseline data and data regarding primary and secondary outcomes will be presented using descriptive statistics. Differences in baseline data between the intervention and control group will be assessed using Student’s *t*-test for continuous data and the *χ*^2^-test for categorical data. A p-value ≤ 0.05 will be considered statically significant.

#### Primary study parameter

For the primary outcome measure (RTW), the number of days until RTW (either full or partial) will be analysed using a Kaplan-Meier survival method. The patients who dropped out of the study will be censored. The differences between the intervention and the control group will be tested using a log rank test. If significant differences between the intervention and control groups are found with regard to the prognostic variables, the outcome variable RTW will be adjusted for these variables in a multivariate Cox regression analysis. In addition to the ITT analysis aimed at ‘all—cause return to work’, the analysis will be performed excluding those patients who died during follow-up and those who have a life expectancy of less than a few months, because they will not RTW.

#### Secondary study parameters

The relative risk and 95 % confidence interval for returning to work (either full or partial) at twelve months of follow-up will be calculated for the intervention group versus the control group. Longitudinal multilevel analysis will be used to examine differences between the intervention and control groups with regard to the secondary outcomes (quality of life, work ability and work limitations).

#### Economic evaluation

The direct and indirect costs will be calculated for each participant. Using bootstrapping, mean differences in direct, indirect, and total costs will be calculated between the control group and the intervention group [43]. Incremental cost-effectiveness ratios will be calculated by assessing the ratio of the differences in costs between the intervention and control groups to the differences in RTW rates between the groups.

## Discussion

The aim of this study is to evaluate the cost-effectiveness of a tailored work-related support intervention for GI cancer patients, which will be carried out as an RCT compared with usual care.

Although some GI cancer patients indicate that they would prefer to continue working even during treatment [1, 22, 44], at present there are no effective work-related interventions for GI cancer patients early in the treatment process [17, 21]. Previous research suggests that interventions should be developed to support patients with work-related problems [44, 45]. The tailored intervention described in this study will provide work-related support at an early stage of the cancer diagnosis and treatment process. In the course of the tailored work-related support intervention, patients will receive individualized support based on their specific work-related problems.

The nurses in this study will receive training prior to the start of the GIRONA study, to enable them to give supportive care to patients in the ‘support A’ intervention group. It is therefore conceivable that bias might occur in this study, because the nurses will become aware of the importance of work for cancer patients. These nurses also provide the usual care in the control group. Due to the nurses’ awareness of the importance of work, there is a possibility that the trained nurses might unintentionally incorporate their understanding of the importance of work into the counselling sessions they provide for patients receiving the usual care. This could mean that patients in the control group receive work-related advice, or that the nurses influence the patient’s self-efficacy, with the effect that the patients themselves start searching for work-related interventions. To monitor this, patients will be asked in the questionnaires whether they have attended additional work-related care sessions. There is a risk, however, that because of the mechanisms described above, the contrast between the intervention group and the control group could be diminished.

Multidisciplinary cooperation within the clinical setting is centred around the patient; this is important when there are complex problems at hand, including work-related problems. For support type C, a multidisciplinary team meeting forms part of the intervention. With this type of support, multiple forms of expertise are combined for the patient’s benefit, in order to prevent contradictory advice from being given to the patients. Providing tailored work-related support within a clinical setting makes it easier for the different healthcare professionals to be in contact with each other. Previous research has shown that information-sharing between healthcare professionals is an essential aspect of effective transition in cancer care [46].

The fact that the oncological occupational physician is working within a clinical setting will ensure that cooperation between the oncological occupational physician and primary care is optimized. This is important, as both are key to a successful RTW.

This study will provide us with information about the effectiveness of tailored work-related support on the RTW during the clinical process, as compared with patients who get the usual care. The intervention will bring a new focus to occupational revalidation early in the treatment process. To date, questions about the RTW have not routinely been addressed early in the treatment process.

If this intervention proves effective, it can be implemented in practice to support GI cancer patients, starting at the moment of diagnosis and in the treatment process, to provide them with support on work-related problems and enhance the RTW. The intervention is innovative in that oncological and occupational care are combined in a clinical setting in the cancer treatment process. The subsequent step will be to apply the intervention to other cancer diagnoses.

The results of the GIRONA study will be available in 2017.

### Ethics approval

The Medical Ethical Committee of the Academic Medical Center Amsterdam, the Netherlands, approved the study. The local medical ethics committees of the participation hospitals gave positive advice on the local feasibility of the study

### Consent for publication

Not applicable

### Availability of data

This article has used no dataset. Therefore no additional data files are available.

### Trial status

Study is currently recruiting patients. First patient was included at June 2015.

## Abbreviations

GI: gastrointestinal
RTW: return to work
RCT: randomized controlled trial
AMC: academic medical center
GIRONA: gastrointestinal cancer patients receiving occupational support near and after diagnosis
